# Dietary niche partitioning in Early Jurassic ichthyosaurs from Strawberry Bank

**DOI:** 10.1111/joa.13744

**Published:** 2022-09-29

**Authors:** Sarah Jamison‐Todd, Benjamin C. Moon, Andre J. Rowe, Matt Williams, Michael J. Benton

**Affiliations:** ^1^ Department of Earth Sciences University College London London UK; ^2^ Bristol Palaeobiology Group, School of Earth Sciences University of Bristol Bristol UK; ^3^ Bath Royal Literary and Scientific Institution Bath UK

**Keywords:** dietary niche partitioning, finite element analysis, ichthyosaurs, Jurassic, marine reptiles, Mesozoic

## Abstract

Jurassic ichthyosaurs dominated upper trophic levels of marine ecosystems. Many species coexisted alongside each another, and it is uncertain whether they competed for the same array of food or divided dietary resources, each specializing in different kinds of prey. Here, we test whether feeding differences existed between species, applying finite element analysis to ichthyosaurs for the first time. We examine two juvenile ichthyosaur specimens, referred to *Hauffiopteryx typicus* and *Stenopterygius triscissus*, from the Strawberry Bank Lagerstätte, a shallow marine environment from the Early Jurassic of southern England (Toarcian, ~183 Ma). Snout and cranial robusticity differ between the species, with *S. triscissus* having a more robust snout and cranium and specializing in slow biting of hard prey, and *H. typicus* with its slender snout specializing in fast, but weaker bites on fast‐moving, but soft prey. The two species did not differ in muscle forces, but stress distributions varied in the nasal area, reflecting differences when biting at different points along the tooth row: the more robustly snouted *Stenopterygius* resisted increases or shifts in stress distribution when the bite point was shifted from the posterior to the mid‐point of the tooth row, but the slender‐snouted *Hauffiopteryx* showed shifts and increases in stress distributions between these two bite points. The differences in cranial morphology, dentition and inferred stresses between the two species suggest adaptations for dietary niche partitioning.

## Graphical abstract

Ichthyosaurs were important marine predators during the Mesozoic, but it has been uncertain whether they all hunted the same prey or not. Here, we explore dietary adaptation by a first application of a numerical biomechanical analysis. *Stenopterygius* specialized in slow biting of hard prey and *Hauffiopteryx* specialized in fast, but weaker bites on fast‐moving, but soft prey. So, these two ichthyosaurs shared out food, thick‐scaled fishes and ammonites for one, fast fish and squid for the other.

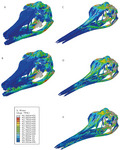

## 1 INTRODUCTION

Ichthyosaurs played an important role as apex predators in Mesozoic oceanic ecosystems (Foffa et al., 2018; Motani, 2005; Reeves et al., 2021; Stubbs & Benton, 2016; Thorne et al., 2011). Ichthyosaurs originated and diversified from 249 Ma, around 3 Ma after the end‐Permian mass extinction event, and their fossil record spans about 160 Ma (Cleary et al., 2015; Motani, 2005; Stubbs & Benton, 2016; Thorne et al., 2011). Partitioning of food resources, hunting modes and swimming styles, and oceanic sub‐habitats allowed for the diversification of these apex predators into a variety of dietary and life mode preferences (Böttcher, 1989; Bürgin, 2000; Dick et al., 2016; Massare, 1987; Pollard, 1968; Reeves et al., 2021; Stubbs & Benton, 2016). Triassic ichthyosaurs exhibited a wide range of body plans, swimming styles and dentition and feeding modes (Gutarra et al., 2019; Massare & Callaway, 1990). Ichthyosaurs passed through a morphological bottleneck after the end‐Triassic extinction ~201 Ma which reduced the breadth of their ecological and dietary niche exploitation (Moon & Stubbs, 2020; Stubbs & Benton, 2016; Thorne et al., 2011). They nonetheless recovered and again became dominant and diverse predators until their extinction at the end of the Cenomanian ~95 Ma, during a time of climatic instability and biodiversity shifts in the oceans (Fischer et al., 2016; Reeves et al., 2021).

Jurassic ichthyosaurs all show a thunniform body plan, adapted for pelagic pursuit predation and rapid sustained swimming and diving (Gutarra et al., 2019; McGowan & Motani, 2003; Motani, 2005; Motani et al., 1999; Reeves et al., 2021). This dolphin‐like body form was accompanied by other key features including large orbits associated with diving, a semilunate tail and dorsal fin associated with rapid sustained swimming, and posterior limb reduction (Massare, 1988; McGowan & Motani, 2003; Moon, 2019; Motani et al., 1999). The long and slender rostrum of certain Jurassic ichthyosaurs is highly convergent with extant piscivores such as gharials and river dolphins (Ballel et al., 2019; McCurry, Evans, et al., 2017; McCurry, Walmsley, et al., 2017).

Throughout the Mesozoic, multiple species of ichthyosaurs commonly co‐existed at single localities, sharing their ecospace, but biological and ecological interactions between ichthyosaur species are poorly understood. Did these top predators simply hunt whatever prey they could find, including cephalopods, arthropods, fishes and other reptiles and somehow divide the spoils, or did different ichthyosaur species specialize on different kinds of prey? Extensive studies of ichthyosaur feeding include direct evidence from gastric remains (e.g. Böttcher, 1989; Bürgin, 2000; Dick et al., 2016; Jiang et al., 2020; Massare, 1987; Pollard, 1968) and inferences from tooth and mandibular morphology (e.g. Massare, 1987; Reeves et al., 2021). Further, ichthyosaurs evidently showed ontogenetic dietary partitioning, with some species at least switching from an exclusive diet of surface‐swimming fishes to deeper‐dwelling cephalopods as they aged, and with matching changes in dentition and jaw mechanics (Dick et al., 2016). McGowan (1973a), in studying *Ichthyosaurus*, reconstructed the jaw musculature and lever‐arm mechanics, showing the low mechanical advantage of the jaw, but considered substantially different diets between taxa to be unlikely.

Two issues emerge from studies of modern predators, first that it can be hard to identify prey preferences as absolutes through life and through all seasons, and that there can be differences between ecological‐behavioural observations and studies of stomach contents. On the first point, modern terrestrial predators often show substantially overlapping diets, simply feeding opportunistically on whatever they can capture and with preferred prey depending mainly on the body size of the predator (Périquet et al., 2015; Vogel et al., 2016). Sharks on the other hand may appear to feed on similar prey, but analysis of stomach contents and oxygen and nitrogen isotopic values from the tissues can show differences in prey preference: all species may feed on crabs, shrimps, cephalopods and fish, but each species tends to show a distinct preference for one prey clade over the others (Albo‐Puigserver et al., 2015; Yemisken et al., 2019). Cetaceans, the most analogous living modern group to ichthyosaurs, also occupy a variety of dietary and trophic niches related to feeding mode and prey preference within a shared ecospace (Weir et al., 2012). Differences between ecological‐behavioural observations and studies of stomach contents are relevant for assumptions about ancient predators. Stomach contents can indicate diets of fossil organisms, but there can be differential survival of skeletal remains, where for example cephalopod hooklets or otoliths may reside in the stomach for a long time, but fish bones are rapidly digested. In a modern example, Fitch and Brownell (1968) found more than 18,000 otoliths in the guts of 17 whales, evidence of massive concentration by ingestion. Such excellent survival and long‐term concentration of stomach contents is suggested by a report (Urlichs et al., 1982) of a 1.5‐m‐long Holzmaden shark with about 250 belemnite rostra in the stomach area. The point is that such examples prove that the predator was eating this particular prey item, but the differential survival of stomach remains means palaeontologists must be cautious in inferring that this was, for example, the sole dietary item.

Here, we compare a juvenile *Stenopterygius triscissus* and a juvenile *Hauffiopteryx typicus* from the Early Jurassic Strawberry Bank locality in southern England. Given the diverse prey animals preserved alongside these ichthyosaurs (Caine & Benton, 2011; Williams et al., 2015), we test whether there is any evidence for dietary niche partitioning. We perform finite element analysis on an ichthyosaur for the first time to test rigorously whether jaw function differed between these two species.

## 2 MATERIALS AND METHODS

### 2.1 Specimens

The now‐inaccessible Strawberry Bank locality of Ilminster, England, preserves a wide variety of specimens representative of an Early Jurassic (Toarcian, ~183 Ma) shallow marine environment (Caine & Benton, 2011; Pierce & Benton, 2006; Williams et al., 2015). During the Early Jurassic, the locality lay near the western continental coast of the Tethys Ocean. The specimens are often fully or partially articulated as they are preserved three‐dimensionally in carbonate nodules, preventing diagenetic flattening of the material. The locality represents a diverse ecosystem, with the remains of multiple species of fish, marine crocodilians, cephalopods, crustaceans and other marine invertebrates, and insects from the nearby continent (Williams et al., 2015). The deposit correlates with the Toarcian Oceanic Anoxic Event, a time of biotic turnover and climate instability driven by major volcanic eruptions of the Karoo‐Ferrar Igneous Province, increased weathering and disruption of ocean circulation (Maxwell & Vincent, 2016; McElwain et al., 2005; Them II et al., 2017; Williams et al., 2015).

**FIGURE 1:**
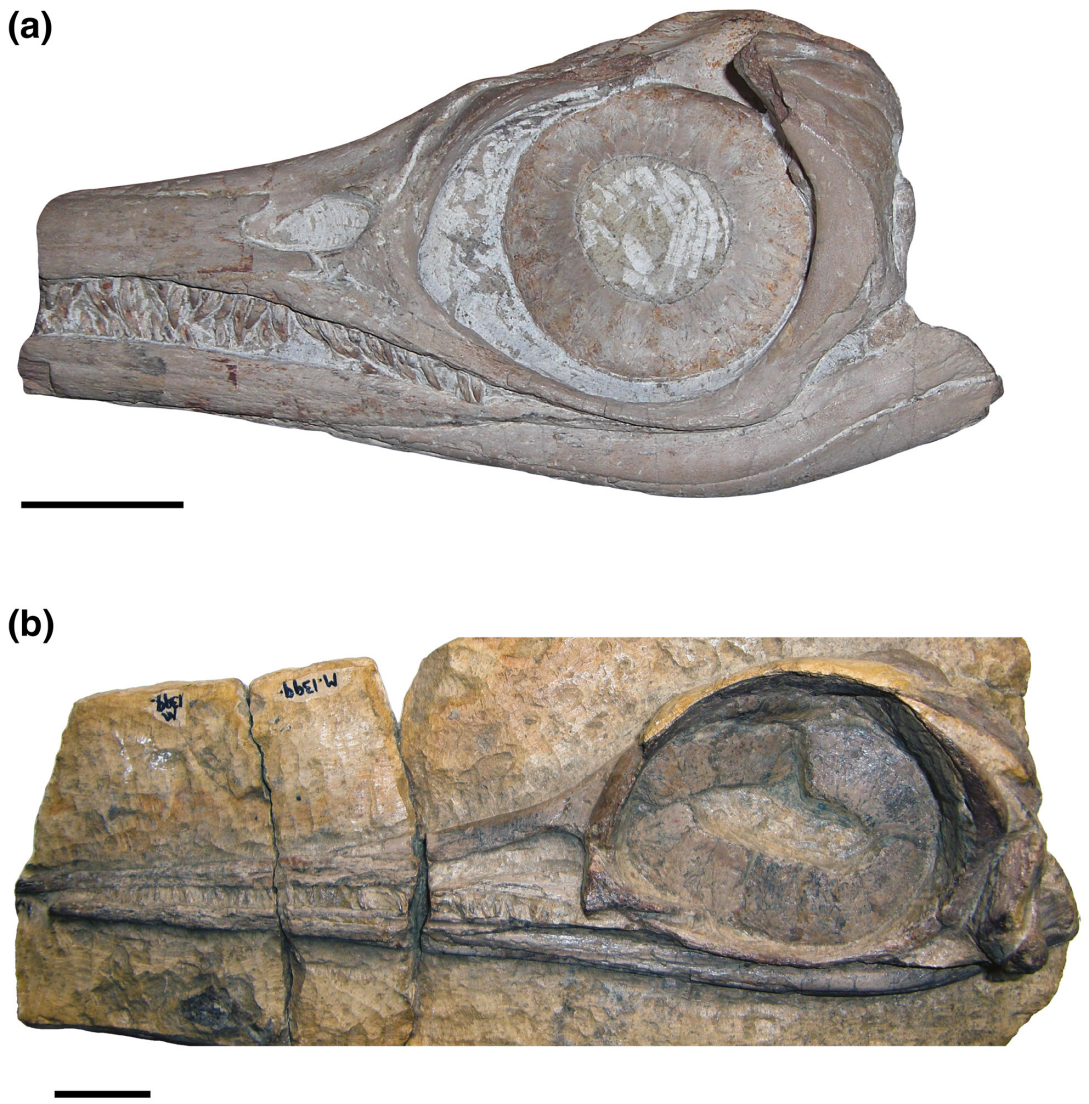
Specimens of two juvenile ichthyosaurs included in this study from Strawberry Bank (Toarcian, Lower Jurassic) of Ilminster, UK. (a), *Stenopterygius* triscissus (BRLSI M1409). (b), *Hauffiopteryx typicus* (BRLSI M1399). Scale bars equal to 30 mm

The Lagerstätte was discovered by Charles Moore in the 1840s, who collected many specimens, including eight juvenile ichthyosaurs (Caine & Benton, 2011; Williams et al., 2015). Ichthyosaurs from this locality are exclusively juveniles belonging to the species *S. triscissus* (Quenstedt, 1858) and *H. typicus* (von Huene, 1931). The concentration of young animals suggests that this was possibly a nursery area, by analogy with certain extant sharks whose juveniles live in sheltered nearshore, shallow marine environments and shift to open‐ocean, pelagic life modes on reaching maturity (Caine & Benton, 2011). The skulls of the two species are broadly similar (Figure 1), except for differences in proportions: *H. typicus* has a more gracile snout than *S. triscissus*, *H. typicus* has a larger orbit than *S. triscissus* and the teeth of *S. triscissus* are larger, more closely packed and more robust than in *H. typicus* (Caine & Benton, 2011). Potential prey animals such as small fish and cephalopods are also found at this locality, and differences in dentition between the two species, with *S. triscissus* having larger, more curved teeth relative to the slender, more conical teeth of *H. typicus*, suggest that they might have practised dietary niche partitioning in this relatively limited ecological environment (Caine & Benton, 2011).

**FIGURE 2:**
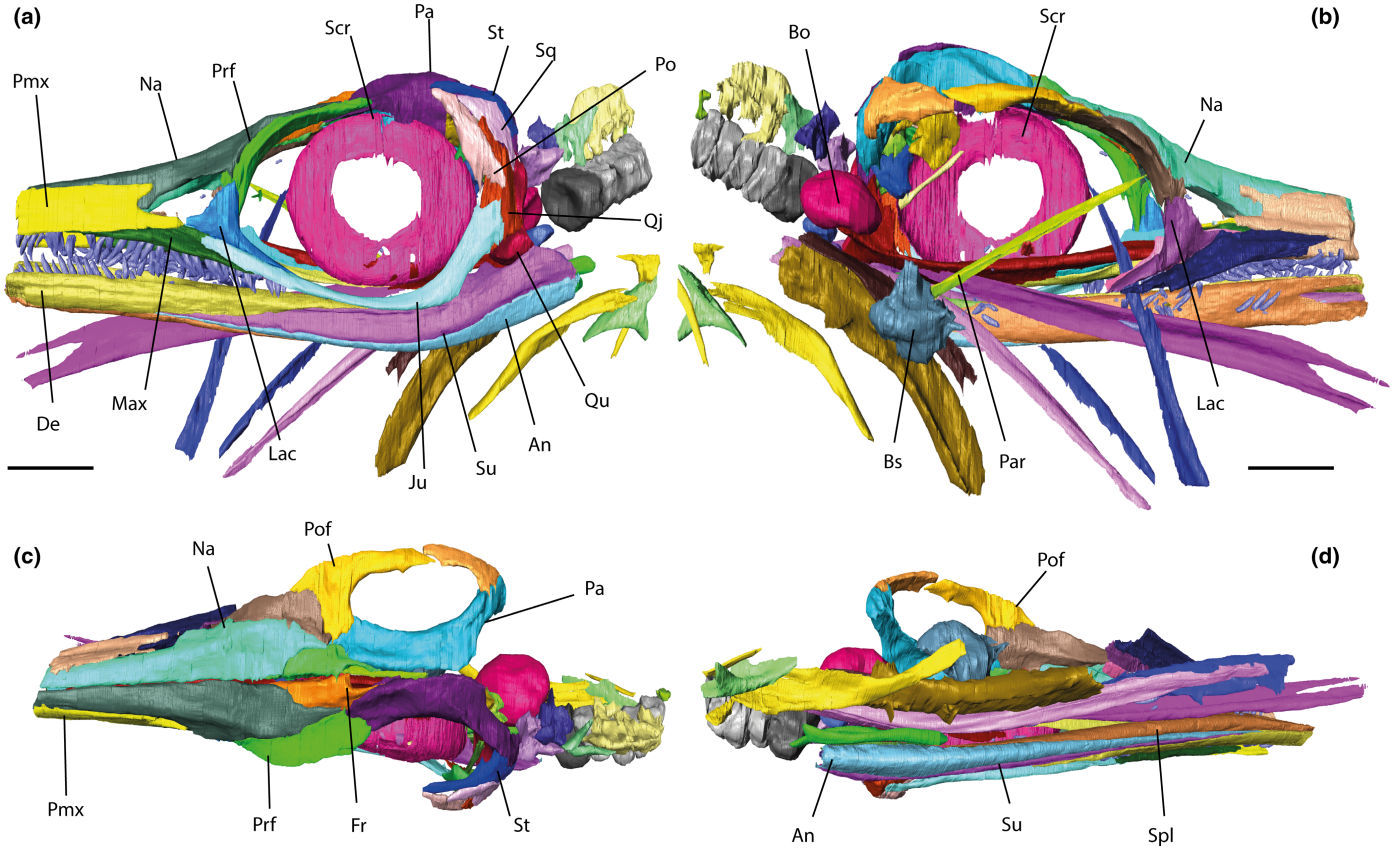
Segmented material of *Stenopterygius triscissus* (BRLSI M1409) prior to reconstruction. (a), left lateral view of *S. triscissus*. (b), right lateral view of *S. triscissus*. (c), dorsal view of *S. triscissus*. (d), ventral view of *S. triscissus*. Abbreviations: An, angular; Bo, basioccipital; Bs, basisphenoid; De, dentary; Ju, jugal; Lac, lacrimal; Max, maxilla; Na, nasal; Pa, parietal; Par, parasphenoid; Po, postorbital; Pof, postfrontal; Pmx, premaxilla; Prf, prefrontal; Qj, quadratojugal; Qu, quadrate; Scr, sclerotic ring; Spl, splenial; Sq, squamosal; St, supratemporal; Su, surangular. Scale bars equal to 30 mm. Refer to Figure 4 for reconstructed models

**FIGURE 3:**
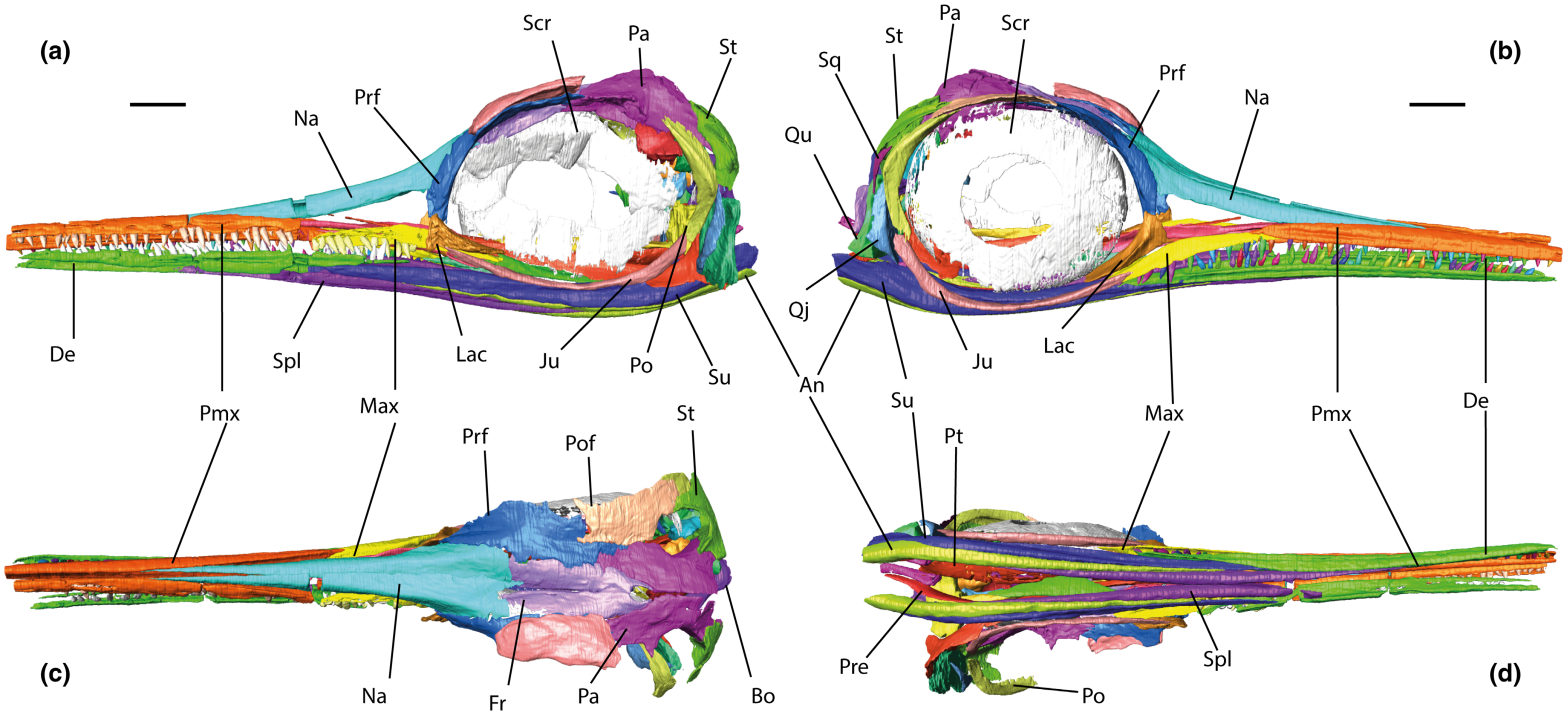
Segmented material of *Hauffiopteryx typicus* (BRLSI M1399) prior to reconstruction. (a), left lateral view of *Hauffiopteryx typicus*. (b), right lateral view of *H. typicus*. (c), dorsal view of *H. typicus*. (d), ventral view of *H. typicus*. Abbreviations: An, angular; Bo, basioccipital; De, dentary; Ju, jugal; lac, lacrimal; max, maxilla; Na, nasal; pa, parietal; Po, postorbital; Pof, postfrontal; Pmx, premaxilla; Pre, prearticular; Prf, prefrontal; Pt, pterygoid; Qj, quadratojugal; Qu, quadrate; Scr, sclerotic ring; Spl, splenial; Sq, squamosal; St, supratemporal; Su, surangular. Scale bars equal to 30 mm. Refer to Figure 4 for reconstructed models

The study specimens are BRLSI M1409 (*S. triscissus*) and BRLSI M1399 (*H. typicus*), both housed at the Bath Royal Literary and Scientific Institution (BRLSI), both preserved three‐dimensionally (Figures 2 and 3). Only the cranial elements are addressed here in the FEA modelling and reconstructions; postcranial elements are not considered beyond the segmentation of the preserved material in the scanned specimens. The preserved cranium of BRLSI M1409 is 185 mm long, while the cranium of BRLSI M1399 is 335 mm long; this length difference is mostly because of the anterior rostrum of BRLSI M1409 is broken off. The total length of the skull in life was estimated as 401 mm for BRLSI M1409 using three examples of exceptionally well‐preserved specimens of *S. triscissus* with complete snouts from Maisch (2008) and the metric of the anteroposterior width of the orbit relative to total skull length. This estimated length places it at the upper end of the range for juveniles of *Stenopterygius* (McGowan, 1979), so it is a large juvenile or subadult.

### 2.2 Computed tomography scanning

BRLSI M1409 was scanned in two parts at the University of Bristol using the Nikon XT H 225 ST μCT scanner. See CT scan image data in Jamison‐Todd et al. (2022). Scan parameters were set to 225 kV, 449 μA, 101 W, 1.5 mm copper filter, 0.5 s exposure time, reflection‐rotating target, 3141 projections and 4 frames per projection. BRLSI M1399 was CT‐scanned at the University of Southampton using a Nikon/Metris 225 kB/450 kV hutch with a 1‐pixel detector. Scan parameters were set to 280 kV, 539 μA, no filter, 160 μm pixel size, 90 ms exposure time, 1701 projections and one frame per projection (Marek et al., 2015).

### 2.3 Segmentation and identification of cranial elements

All cranial elements of both specimens were segmented and identified in Avizo Lite v. 9.7 (FEI Visualization Science Group; https://www.thermofisher.com) (Jamison‐Todd et al., 2022, Avizo files). Each element was assigned to a separate material. BRLSI M1409 was largely articulated on the left side, and BRLSI M1399 was articulated on both sides. BRSLI M1399 had been previously segmented and reconstructed (Marek et al., 2015). Cranial element surfaces were smoothed and exported as STL files for reconstruction in the case of BRLSI M1409 and reconstructed partially in Avizo before being exported in the case of BRSLI M1399. The posterior third of the nasals as well as the anterior postfrontal and squamosal are not preserved in BRLSI M1399, resulting in gaps in the digitally reconstructed skull roof along the prefrontal‐frontal suture, the inter‐frontal suture, the supratemporal and the cheek region, and the prefrontal‐postfrontal suture that would have been covered by the nasal and postfrontal, respectively. These gaps are not expected to influence stress distribution in the rostrum in our digital model. The preservation of M1409 causes a gap between the prefrontal and postfrontal in this specimen, which may influence stresses in this area of the skull roof but is also not expected to influence the rostral stresses.

### 2.4 Reconstruction of the crania

**FIGURE 4:**
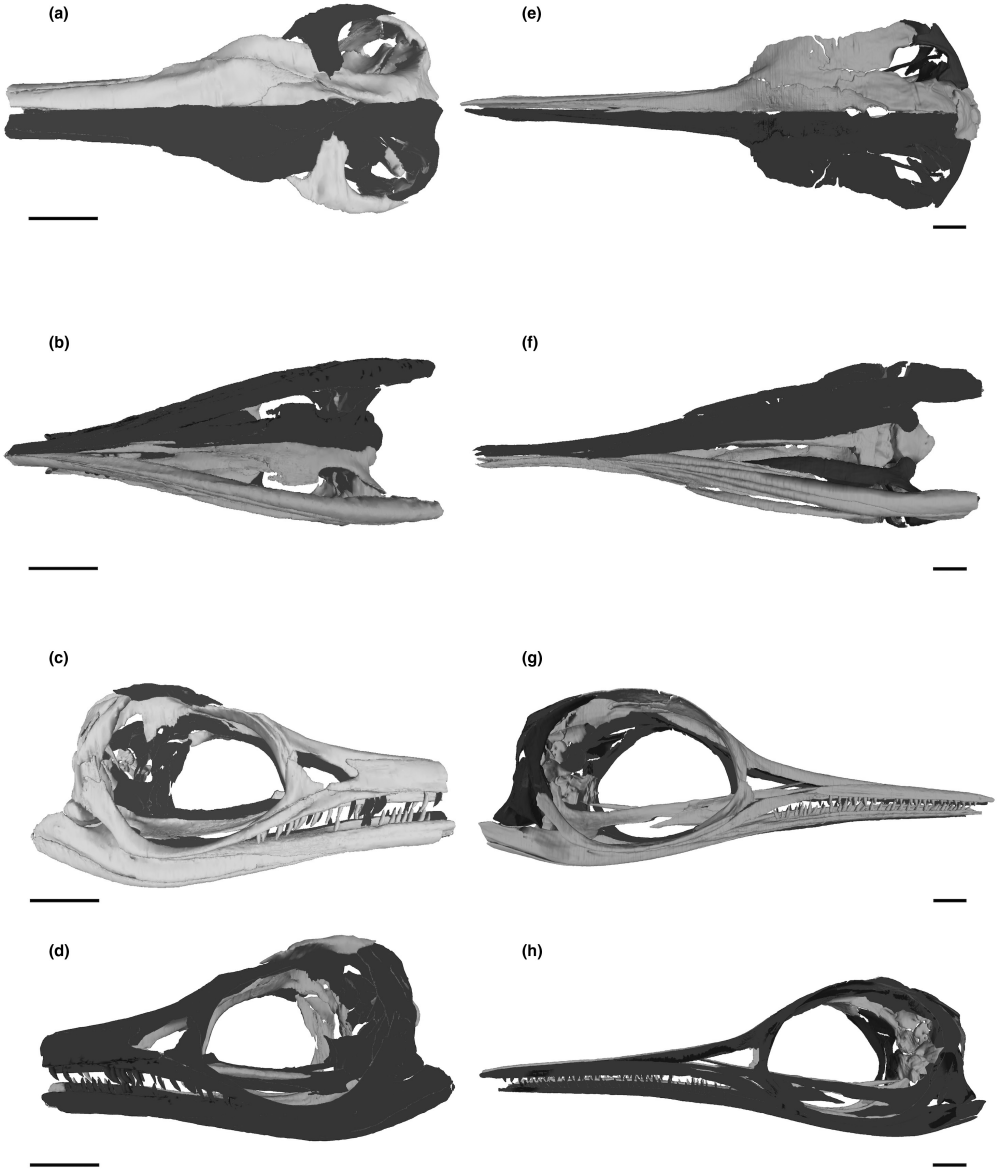
Reconstructed complete skull models of *Stenopterygius triscissus* (BRLSI M1409) and *Hauffiopteryx typicus* (BRLSI M1399). *S. triscissus* in a, dorsal view; b, ventral view; c, right lateral view; d, left lateral view. *H. typicus* in e, dorsal view; f, ventral view; g, left lateral view; h, right lateral view. Darker elements have been mirrored during reconstruction. Scale bars equal to 30 mm

Identification and reconstruction of cranial elements were aided by previous descriptive work illustrating and describing the cranial elements of Jurassic ichthyosaurs and their relative positions in the skull (Marek et al., 2015; Maxwell et al., 2012; McGowan, 1973a; Moon & Kirton, 2016). Cranial elements were reconstructed and mirrored (Figure 4) following the protocols outlined by Lautenschlager (2016).

BRLSI M1399 was reconstructed partially in Avizo by mirroring the left opisthotic, quadrate, supratemporal, squamosal, parietal and postfrontal (Marek et al., 2015). Individual right cranial element surfaces were then exported to Blender v. 2.93 (Blender Institute; http://www.blender.org). The jugal was rearticulated with the quadratojugal and lacrimal, and the quadrate rearticulated with the mandible. The pterygoid was in two pieces and was infilled and smoothed to fuse the element. The articular was missing and was replaced with a duplicate mirrored articular from BRLSI M1409, as this is a closely related taxon, and the rounded disk shape was likely conserved in both. The braincase in both specimens was rearticulated and fitted into the cranium. The right opisthotic and exoccipital were duplicated and mirrored to complete the braincases.

The left cranial elements of BRLSI M1409 are all present except the postfrontal. The reconstruction was done in Blender, with the right postfrontal imported and mirrored to replace the missing left postfrontal. Some of the teeth were articulated and some of the disarticulated teeth were used to fill in notable gaps in the tooth row.

The prootic and stapes were excluded from braincase reconstructions, as the prootic does not attach structurally to the cranium and no identifiable stapes were present. The absence of a stapes potentially alters stress distributions in the crania, but our focus is on relative stress distributions, particularly in the rostrum, and our results should therefore not be affected overmuch. Given that living specimens would have been fully symmetrical, the left cranium of BRLSI M1409 and the right cranium of BRLSI M1399 were duplicated after the reconstruction was complete and mirrored to reduce the effects of diagenetic processes on our results. The braincase reconstructions were then fitted into the crania to complete the skull models (Jamison‐Todd et al., 2022, Blender models).

### 2.5 Muscle reconstruction

For the muscle reconstruction, we followed McGowan's (1973a) detailed account of the origins and insertions of the jaw musculature in *Ichthyosaurus communis*. Muscles were constrained by the cranial elements and the position of other reconstructed muscles, and as rugosity and ridging were difficult to determine on the CT scans, origins and insertions were taken from the descriptions of McGowan (1973a) that he had taken from visible bone surfaces undisguised by sediment, and by homology with living diapsids. Indentations and ridges on the bone were sometimes visible at the muscle attachments in our segmented models, and these were used as a guide in determining the surface area of the attachments. The main areas of insertion and origin can thereby be determined on both skulls from the arrangement and structure of the cranial elements.

**FIGURE 5:**
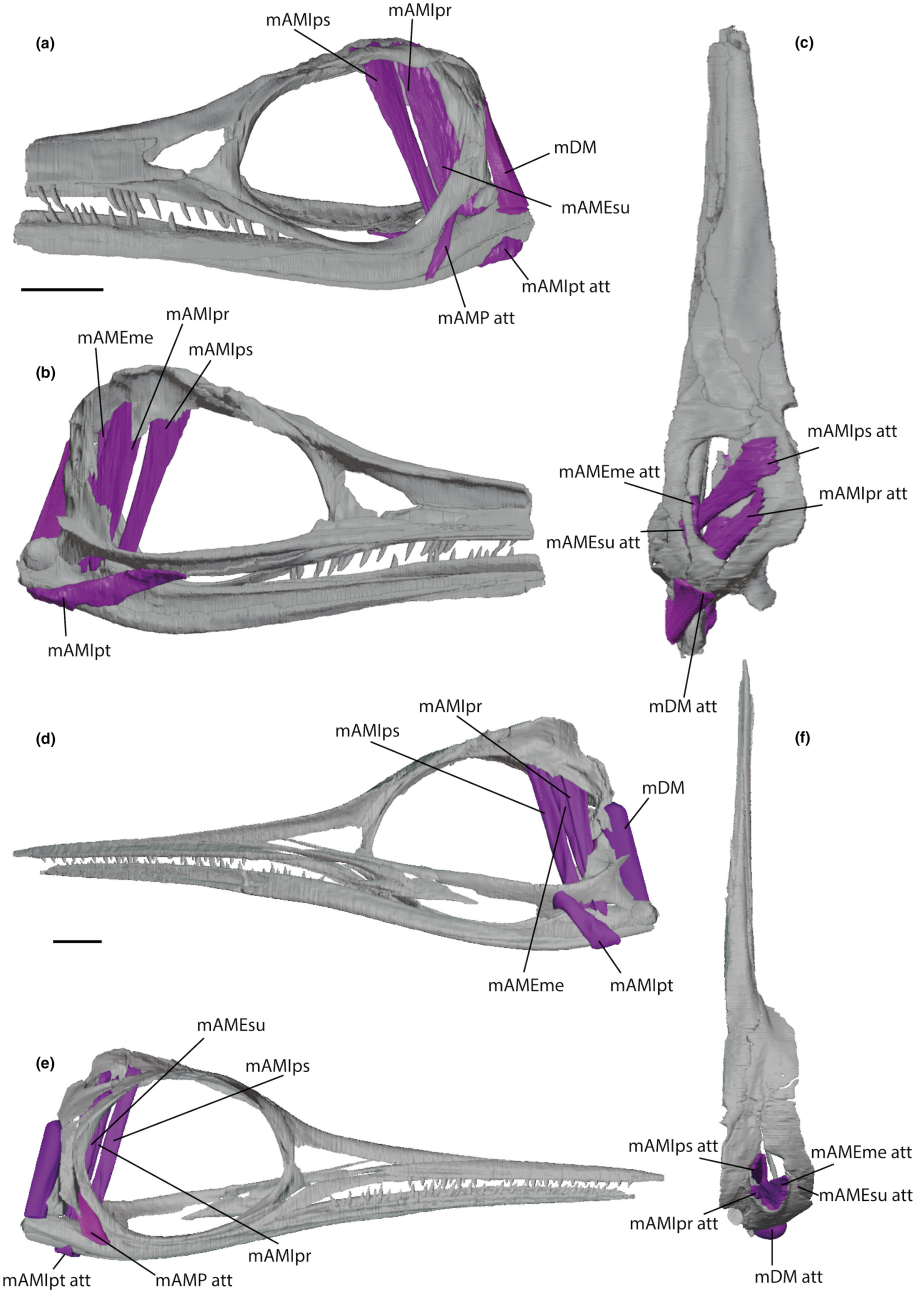
Muscle reconstructions for *Stenopterygius triscissus* (BRLSI M1409) and *Hauffiopteryx typicus* (BRLSI M1399), using one half of the skull. For *S. triscissus*, the left half of the cranium was used, and for *H. typicus*, the right half was used. (a), left lateral exterior view of *S. triscissus*. (b), right lateral interior view of *S. triscissus*. (c), dorsal view of *S. triscissus. (*d), left lateral interior view of *H. typicus*. (e), right lateral exterior view of *H. typicus*. (f), dorsal view of *H. typicus*. Abbreviations: mAMEme, *M. adductor mandibulae externus medialis*; mAMEpr, *M. adductor mandibulae externus profundus*; mAMEsu, *M. adductor mandibulae externus superficialis*; mAMIps, *M. adductor mandibulae internus pseudotemporalis*; mAMIpt, *M. adductor mandibulae internus pterygoideus*; mAMP, *M. adductor mandibulae posterior*; mDM, *M. depressor mandibulae*. “att” qualifier indicates muscle attachment areas. Scale bars equal to 30 mm

We follow the procedure described by Lautenschlager  (2013) for digital, three‐dimensional muscle reconstruction and we used Avizo. This procedure creates flat, linear muscles that fill the minimal volume required to connect the muscle attachment areas to one another. This may provide an underestimate of muscle volume and therefore muscle force magnitudes, whereas filling in the muscles outside the linear bounds of the attachment areas and extending to fill the cranial volume may provide an overestimate. Either method will produce similar stress distributions on the crania, with the magnitude of the forces differing, but this should not affect our assessment of comparative biomechanical function. Seven separate muscle divisions associated with the opening and closing of the jaws were created by identifying origins and insertions of the muscles and segmenting between those areas (Table 1). The *M. adductor mandibulae externus* group was divided into three: *M. adductor mandibulae externus superficialis* (mAMEsu), *M. adductor mandibulae externus medialis* (mAMEme), and *M. adductor mandibulae externus profundus* (mAMEpr). The *M. adductor mandibulae internus* group was divided into two: the *M. adductor mandibulae internus pseudotemporalis* (mAMIps) and *M. adductor mandibulae internus pterygoideus* (mAMIpt). The *M. adductor mandibulae posterior* (mAMP) and *M. depressor mandibulae* (mDM) were also reconstructed (Figure 5) (Jamison‐Todd et al., 2022, Avizo musculature label files).

**TABLE 1:** The seven jaw muscles and their insertions and origins

Muscle	Abbreviation	Origin	Insertion
*M. adductor mandibulae externus profundus*	mAMEpr	Posterior internal rim of temporal vacuity	Coronoid process
*M. adductor mandibulae externus superficialis*	mAMEsu	External rim of temporal vacuity	Coronoid process
*M. adductor mandibulae externus medialis*	mAMEme	External rim of temporal vacuity	Coronoid process
*M. adductor mandibulae internus pseudotemporalis*	mAMIps	Anterior internal rim of temporal vacuity	Coronoid process
*M. adductor mandibulae internus pterygoideus*	mAMIpt	Pterygoid flange	Wraps around posterior of mandible on angular
*M. adductor mandibulae posterior*	mAMP	Anterior of quadrate	External surface of mandible behind jugal
*M. depressor mandibulae*	mDM	Posterior supratemporal	External posterior surface of surangular

*Note*: Insertions and origins were determined from McGowan (1973a) and from visible attachment areas on the crania.

Muscle lengths were measured in Avizo as the longest line through the reconstructed muscle (Table 2). Muscle volumes were measured using an Avizo statistics module and from length measurements and adductor chamber dimensions, which were taken from the length and width of the top of the segmented adductor chamber models to provide an estimated total volume by the approximation of a square‐based pyramid (Table 2). This was done for a control comparison of total muscle volume and average length.

**TABLE 2:** The dimensions and calculated forces produced by each of the seven reconstructed muscles for BRLSI M1409 and M1399

	M1409 *Stenopterygius triscissus*				M1399 *Hauffiopteryx typicus*			
Muscle	Length (mm)	Volume (mm^3^)	Cross‐sectional area (mm^2^)	Muscle force (N)	Length (mm)	Volume (mm^3^)	Cross‐sectional area (mm^2^)	Muscle force (N)
mAMEpr	72	2085	87	26	83	1556	56	17
mAMEsu	60	1599	80	24	62	2515	122	37
mAMEme	62	1227	59	18	77	2094	82	24
mAMIps	73	1461	60	18	85	2660	94	28
mAMIpt	55	1648	90	27	37	1591	129	39
mAMP	54	794	44	13	54	1950	108	33
mDM	45	1595	106	32	57	7214	380	114

*Note*: The forces represent the total muscle force on an attachment area prior to division by the number of nodes and using total length divided by 3 for the maximum length of a muscle fibre.

### 2.6 Finite element analysis

Finite element analysis (FEA) is a method to test stress and strain distributions on three‐dimensional objects of complex geometry, and in palaeobiology is most often applied to the reconstructed skeletal elements of extinct organisms to determine biomechanical function (Rayfield, 2007). Many studies have combined muscle reconstruction with reconstructed cranial models to determine bite force and feeding mode and ability, thereby making inferences about the ecology and diet of extinct organisms (Ballel et al., 2019; Button et al., 2016; Endo et al., 2002; Lautenschlager, 2015; Lautenschlager et al., 2013; McCurry, Walmsley, et al., 2017; Rayfield, 2007; Ross et al., 2005; Taylor et al., 2017).

Comparative studies of two or more organisms are the best use of the FEA method; this can avoid uncertainties over estimates of reconstructed stresses by following identical protocols for all models, so that any differences in outcomes may reveal true differentiation in function (Ballel et al., 2019; Button et al., 2016; Dumont et al., 2005, 2009; Endo et al., 2002; Lautenschlager, 2015; Lautenschlager et al., 2016; McCurry, Walmsley, et al., 2017; Rayfield, 2007; Taylor et al., 2017). Computational models such as FEA are non‐invasive and provide an opportunity for complex force response testing in three‐dimensional models (Dumont et al., 2009; Rayfield, 2007). A three‐dimensional cranial model is divided into a mesh of tiny elements for detailed observation of stress response throughout the structure, and parameterization of the model includes the input of material properties, most often bone or muscle, and the assignment of force magnitude and direction based on the length, size and position of the reconstructed cranial elements and musculature (Rayfield, 2007). We assigned only the material properties of bone to the models, discounting potentially cartilaginous elements and elements that might have been unfused in young specimens (Miedema & Maxwell, 2022). We cannot identify the ontogenetic stage of the specimens other than to say they are juveniles, but the cartilaginous elements in a juvenile skull would spread the forces rather than alter their general distribution, which is the focus of this study. Flexibility of sutures was also not considered as the ichthyosaurian skull was likely akinetic, as indicated by the interdigitating and overlapping skull sutures (McGowan, 1973a). This parameterization created slightly simplified models that can be used for comparison, especially in the rostra where suture variability and cartilaginous elements are unlikely to have been significant issues. These procedures then enable the calculation of bite forces using lever mechanics. While noting the missing anterior portion of BRLSI M1409, we do not think this will have a significant effect on the FEA reconstructions as the primary resistive structures (e.g. orbit, palate, temporal regions) are present and there is no force applied anterior to each point. Regardless, we have performed an additional analysis of *H. typicus* with the rostrum truncated to match the missing rostrum in *S. triscissus*, to demonstrate the absence of the rostrum in *S. triscissus* has little effect on the analyses (Figure S2).

The models were first downsampled in Blender until the *S. triscissus* model had 702,542 faces and the *H. typicus* model had 718,759 faces. The models were re‐meshed using the voxel re‐mesh and triangulate modifiers to create a more uniform mesh. Remaining non‐manifold mesh errors were fixed by deleting, remeshing and smoothing small sections of the model surface individually (Jamison‐Todd et al., 2022, .stl and Hypermesh model files). The models of the completed crania were then exported to HyperMesh v14 (Altair Hyperworks; https://altairhyperworks.co.uk). The shrink wrap tool was used to reduce mesh and surface complexity and a tetrahedral 3D mesh of each cranium was created using an element size of 0.5 mm (Bright & Rayfield, 2011). This size is appropriate given the complexity and size of the skull models. The number of elements for the FEA model of *H. typicus* is 1,118,658 and 1,182,291 for *S. triscissus*. Material properties were assigned to the mesh based on extant crocodilian cranial bone, after Ballel et al. (2019). Young's Modulus was assigned a value of 15,000 MPa and Poisson's ratio a value of 0.29.

The standard measured value for muscle stress of 300 kPa in living vertebrates was used in the force calculations for each muscle as follows (Ballel et al., 2019; Rayfield, 2007):

(1) $$F_{\max}=\frac{V}{L}P.$$

This equation provides the maximum force produced by each muscle (*F*_max_) given the muscle volume (*V*) and length (*L*), to produce an average cross‐sectional area, and the muscle stress (*P*) (Table 2). Each muscle length was divided by three to estimate the length of a muscle fibre that can extend up to ⅓ of the total muscle length (Ballel et al., 2019; Bates & Falkingham, 2012). Performing this calculation for each muscle allowed for the assignment of force magnitudes at each set of nodes on the mesh. Nodes were assigned at each muscle attachment of the seven muscles reconstructed.

The number of nodes assigned to each muscle origin and attachment area was decided based on available surface area, and numbers were the same for each loading area in both models. Fifty nodes were assigned at the mAMIps origin and 30 nodes at the mAMEpr origin. The mAMEsu and mAMEme share a common area of origin, and were grouped, with 35 nodes assigned at their origins. These four muscles share a common attachment area and the insertion nodes for this group were combined for a total of 115 nodes. The mAMIpt was assigned 50 nodes, the mAMP 25 nodes and the mDM 40 nodes at origin and insertion (Table 2). The resulting forces were then divided by the number of nodes at each muscle attachment area to determine the force applied to each individual node.

Force vectors were assigned using the “two nodes” method in HyperMesh, with a vector assigned between a node at the origin and attachment of each of the seven jaw muscles on each side of the crania. This ensured that the forces were acting in accordance with the direction of pull of the musculature.

Constraints were applied in both specimens with zero degrees of freedom and ten nodes on each side of the basicranium. Due to the missing portion of the rostrum in *S. triscissus*, two FEA analyses were performed with constraints at the most anterior teeth of the preserved material (approximate middle of the snout) and at the most posterior teeth. In *H. typicus*, three FEA analyses were performed with constraints on the teeth at the tip of the preserved material, at the most posterior teeth, and at the midpoint of the tooth row. The foremost tooth constraints in *S. triscissus* are therefore analogous to the mid‐point bite of *H. typicus*. Five constraint nodes were applied to each tooth or area of the mandible, for a total of 20 nodes at front, back and mid‐point of the tooth row. These constraints were applied symmetrically at both sides of the model to simulate bilateral biting. The models were assigned steps and each set of tooth constraints was separately exported to perform separate analyses based on those constraints. See Jamison‐Todd et al. (2022) fully loaded and constrained HyperMesh files. We also performed an additional analysis on the *H. typicus* model with the rostrum truncated as in *S. triscissus* to affirm that the broken rostrum in *S. triscissus* would not affect the comparison between the two models (see Figure S2).

**FIGURE 6:**
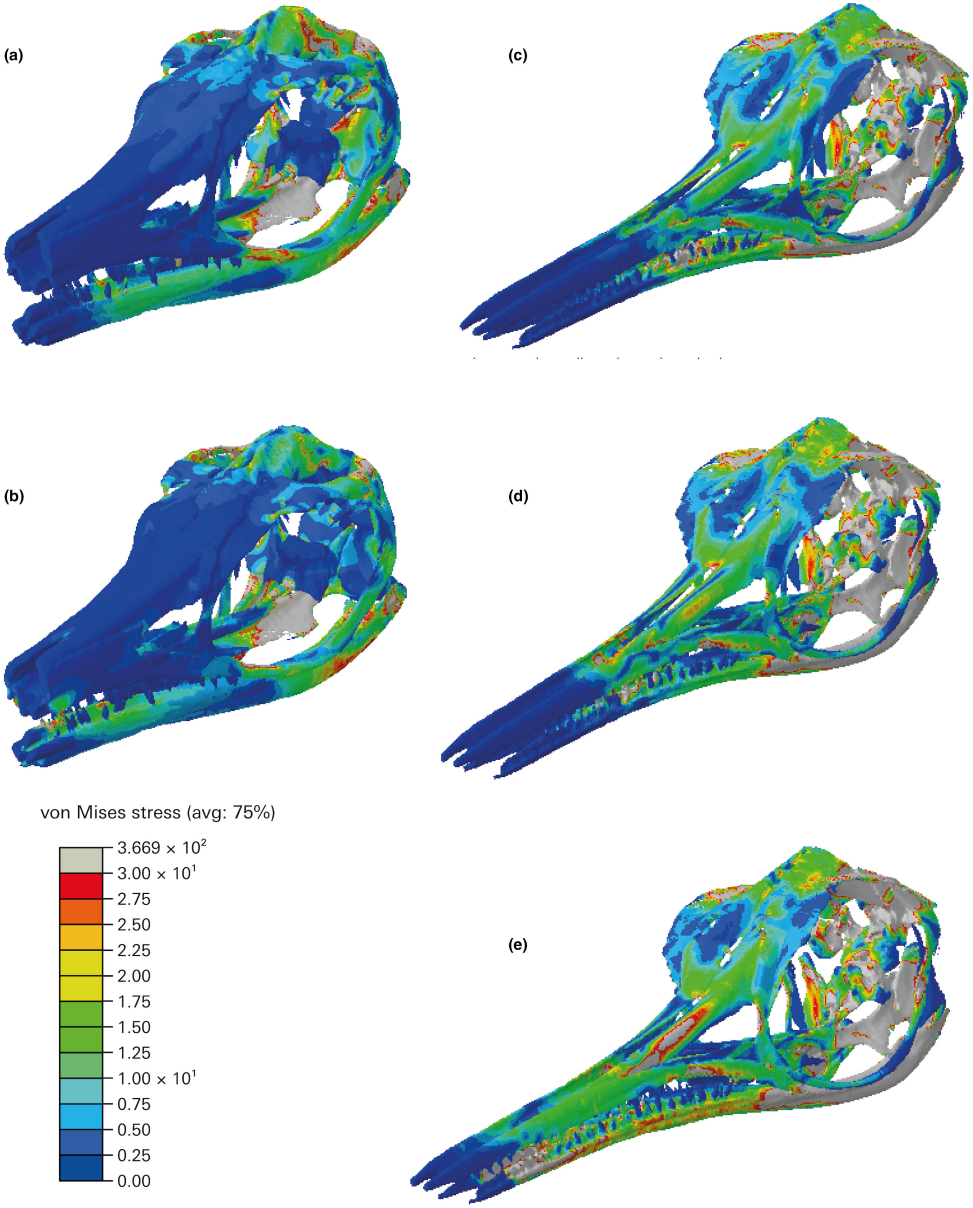
Stress distributions resulting from the FEA. The visible scale is from 0 to 3 MPa, warmer colours indicating higher stress. Note the differences in stresses on the rostrum between the two species in particular and overall higher stresses in the more gracile cranium of *Hauffiopteryx typicus* than in *Stenopterygius triscissus*. (a), *S. triscissus* (BRLSI M1409) with a simulated bite at the most posterior teeth. (b), *S. triscissus* with a simulated bite at the anterior of the preserved material of the tooth row. (c), *H. typicus* (BRLSI M1399) with a simulated bite at the most posterior teeth. (d), *H. typicus* with a simulated bite at the mid‐point of the tooth row. (e), *H. typicus* with a simulated bite at the anterior of the preserved material of the tooth row. The anterior bite point of *S. triscissus* and the mid‐point bite of *H. typicus* are analogous

FEA was performed in Abaqus v. 6.14 (Dassault Systèmes Simulia; https://www.3ds.com). A contour map was generated for each model showing the von Mises stress distributions in the crania as an indicator of resistance to compressive and tensile stress failure (Rayfield, 2007; Dumont et al., 2009; Jamison‐Todd et al., 2022, Abaqus models) (Figure 6). The von Mises scale was adjusted to show values between 0 and 3 MPa on all models for the best comparison and visual representation of the stress distributions.

### 2.7 Relative bite force

The mechanical advantage of each specimen was calculated as the length ratio of the in‐lever to the out‐lever. The distance from the rear of the mandible to the muscle attachments at the jaw articulation is the in‐lever and the total length of the jaw is the out‐lever; this ratio provides a metric for biting ability (Anderson et al., 2011; Ballel et al., 2019; McGowan, 1973a). The preserved material of BRLSI M1409 of 185 mm was projected to 401 mm relative to the complete 335 mm of BRLSI M1399 (Figure S1). The total projected length of the rostra was used to calculate bite force (*F*) using the equation:

(2) $$F=F_{\max}A$$

or the mechanical advantage (*A*) multiplied by the maximum muscle force (*F*_max_) (Ballel et al., 2019; McGowan, 1973a).

## 3 RESULTS

### 3.1 Morphological distinctions

From observation prior to reconstruction and modelling, some morphological differences between the two specimens are immediately apparent (Figure 1). *H. typicus* has a large cranial area relative to the rostrum, with a generally more gracile skull, larger eye orbit and a more slender rostrum, whereas *S. triscissus* has a more robust skull with a longer rostrum relative to the rest of the cranium. The teeth of *S. triscissus* are longer and more densely packed than the smaller, more conical teeth of *H. typicus* (Figure 1). There are no gaps between the teeth of *S. triscissus*, while *H. typicus* has gaps of 1–3 mm between teeth where the tooth rows are complete. Ten measurements of tooth length and width across the widest point of the crown on each specimen produced an average aspect ratio of 0.3 for *S. triscissus* and 0.4 for *H. typicus* (Table 3).

**TABLE 3:** Tooth measurements and aspect ratios for both specimens

	M1409 *Stenopterygius triscissus*			M1399 *Hauffiopteryx typicus*		
	Length (mm)	Diameter (mm)	Aspect ratio	Length (mm)	Diameter (mm)	Aspect ratio
	11.9	2.2	0.2	7.3	3.3	0.5
	10.2	4	0.4	6.8	2.3	0.3
	9.5	2.4	0.3	9.8	2.7	0.3
	8.8	1.6	0.2	5.3	2	0.4
	9.5	1.8	0.2	8.8	3.7	0.4
	10.5	1.7	0.2	7.2	2.8	0.5
	9	2	0.2	7.4	2.4	0.3
	8.4	5.6	0.7	6.3	2.1	0.3
	7	1.9	0.3	4.9	2.3	0.5
	11.1	2.6	0.2	8.4	2.5	0.3
Mean	9.6	2.6	0.3	7.2	2.7	0.4

### 3.2 Muscle reconstruction distinctions

The complete volume of the seven jaw muscles reconstructed here is approximately equivalent to estimated volumes of the adductor chamber in each specimen. This shows that the total calculated muscle volume is in line with cranial morphology, and though the geometry used for reconstructions and estimates as outlined in our methods might lead to an underestimate of total muscle force, the relative stress patterns should remain the same even if the force magnitudes are lower than they might have been in life. The average length of the muscles of *S. triscissus* from origin to insertion is 60 mm, while the jaw muscles of *H. typicus* average 65 mm in length, reflecting the larger size of the specimen. The total muscle volume of *S. triscissus* is 10,409 mm^3^ and of *H. typicus* 19,581 mm^3^ (Tables 2 and 4).

**TABLE 4:** Total muscle volumes, mechanical advantages, muscle forces and relative bite force of both specimens

Measurement	M1409 *Stenopterygius triscissus*	M1399 *Hauffiopteryx typicus*
Total muscle volume (mm^3^)	10,409	19,581
Mechanical advantage	0.089	0.191
Total muscle force (N)	158	291
Bite force at tip of tooth row (N)	14	56
Bite force at back of tooth row (N)	68	181

*Note*: These values are all much greater in *H. typicus* despite the greater resistance of *S. triscissus* to stresses on the cranium. There is no unit for mechanical advantage.

### 3.3 Stress distribution on the crania

The FEA results show somewhat comparable stress distributions across both crania, but with some important differences (Figure 6). Stresses around the posterior and dorsal areas of the crania surrounding the temporal fenestra are higher at the muscle attachment areas on both crania. Other areas of high stress are the mandibular muscle attachment areas, and the highest levels of stress in the palate area are expressed in the pterygoid at the muscle attachments, with the rest of the palate less affected. Stresses on the palate area are only interpreted as relative to the muscle force at the attachment areas, as the palate reconstructions between the two specimens have some differences and the rostral stresses are the focus of this study. *H. typicus* shows overall higher and more widely distributed stresses than *S. triscissus*, particularly in the nasal, lacrimal and premaxilla areas. The mid‐tooth row bite simulation of *H. typicus* is comparable to the mid‐tooth row bite simulation of *S. triscissus* at the anterior of the preserved material. Stress along the dorsal surface of the nasal bone is highest in these mid‐point simulations and is much higher in the more gracile rostrum of *H. typicus*; the nasal area produces the greatest differences in stress distributions between the two species. The cranium of *H. typicus* shows greater shifts in stresses between assigned bite points, while the cranium of *S. triscissus* is only slightly affected by the shift from a posterior to middle bite point. The middle bite point of *H. typicus* produces the highest nasal stress.

### 3.4 Biting mechanics

The calculated bite force at the tip of the tooth row based on two‐dimensional lever mechanics using the total length of 335 mm for BRLSI M1399 and the estimated total length of 401 mm for BRLSI M1409 is 14 N in *S. triscissus* and 56 N in *H. typicus*; the calculated bite force at the back of the tooth row is 68 N in *S. triscissus* and 181 N in *H. typicus* (Table 4). The difference in bite forces results from factors such as total muscle volume and mechanical advantage being significantly higher in *H. typicus*. As a point of comparison, the force at a single node representing the bite force at the tip of the tooth row in the model of *H. typicus* was obtained from the FEA analysis. This bite force estimate of 59 N is similar to the two‐dimensional lever mechanics calculation of 56 N for the tip of the tooth row in *H. typicus*, confirming that both values may be plausible.

## 4 DISCUSSION

### 4.1 Ichthyosaur feeding ability from biomechanical analysis

We present the first reconstructions of muscle force and biting mechanics in an ichthyosaur. Although similar in size, the total muscle volume, mechanical advantage, total force acting on the cranium from the muscles, and bite force at the tip of the tooth row are all much greater in *H. typicus* than in *S. triscissus* (Table 4). Despite smaller temporal fenestra and muscle attachment areas in *H. typicus*, the differences in muscle volume likely arise from the differing proportions of the skull. *H. typicus* has a taller cranium relative to the length of the rostrum, with the additional volume filled by muscles, and thus generating higher overall muscle force. This is more effectively utilized through higher mechanical advantage generating a higher force relative to the biting point at the tip of the tooth row in *H. typicus* than in *S. triscissus*.

The FEA results show that the stress on the nasal area of the rostrum differs most between the two species, and the robusticity of the rostrum is therefore important (Figure 6). The higher and more widely distributed stresses on the cranium of *H. typicus* relative to *S. triscissus* show that the former was less resistant to feeding stresses, in the nasal area in particular, and would have been less adapted for crushing food despite its higher muscle forces. We emphasize the difference in stresses on the rostrum because these are most relevant to biting ability. It is intuitive to assume that the stresses on the more gracile cranium of *H. typicus* would be higher, especially with the greater overall muscle volume, and the general stress patterns on the braincase and skull roof show this. Regardless of differences in stress magnitudes, the general patterns of stress across the two crania are similar (Figure 6). The shift in bite point along the tooth row barely affects the stresses on the cranium of *S. triscissus*, supporting the conclusion that it was able to bite effectively at any point on the tooth row without stressing the rostrum greatly and therefore would have been better adapted for the tearing and sustained biting required by larger prey and supported by the robust, slightly curved dentition. *H. typicus* is more sensitive to these shifts in bite point and has a cranial morphology less resistant to the stresses produced by the muscle forces. However, the largest changes in stresses and relative shifts in stress distribution as seen in the rostrum are the focus of our conclusions regarding biting ability between the two specimens.

These new findings corroborate the morphology of the skulls. The dentitions differ, with dense, robust teeth in *S. triscissus* and shorter, conical teeth in *H. typicus*, suggesting that the former was better adapted for crushing or tearing prey, and may have been hunting larger fish or squid, while *H. typicus* specialized in smaller fish and softer prey. Ichthyosaurs in general, as apex predators with feeding modes requiring resistance to tearing and crushing forces, tend to have larger, more curved teeth, while shorter and more conical teeth indicate a generalist or soft‐prey diet (Fischer et al., 2016; Massare, 1987). The former is associated with a more robust rostrum, while the latter is associated with a more slender rostrum, as seen in BRLSI M1409 and BRLSI M1399 (Fischer et al., 2016). We confirm that the more gracile skull of *H. typicus* is less fortified against tearing and biting, and in particular that a narrower rostrum is less resistant to torsion. In longirostrine crocodilians, differences in cranial and rostrum robusticity have been determined previously through FEA to make distinctions in diet and feeding mode, and the convergent longirostrine cranial morphology of ichthyosaurs suggests that similar fine‐scale adaptations allowed for differentiation of diets (Ballel et al., 2019).

These differences in morphology and inferred stresses confirm that *H. typicus* was adapted to fast but weak snapping, adapted for prey such as squid or small fish that move fast but have relatively weak skeletons. *S. triscissus*, on the other hand, with a more robust cranium and the teeth of a larger‐prey predator, likely used the force of the jaw muscles for a stronger, more crushing bite that was sustained during feeding, and adapted for slower‐moving prey such as larger fishes, perhaps non‐teleosts with heavier, ganoin‐covered scales.

### 4.2 Diversity of ichthyosaur diets

These findings should be interpreted in the context of wider evidence about ichthyosaur diets. Jurassic ichthyosaurs show relative dental homodonty, unlike the diversity of tooth types in Triassic ichthyosaurs (Massare, 1987; Massare & Callaway, 1990). Stouter and more robust teeth were more common in the Triassic and may have been employed in crushing or chewing hard prey, whereas slender, more elongated or curved teeth may have served as fish traps for prey to be caught and swallowed whole, as seen also in Jurassic ichthyosaurs (Massare, 1987; McGowan & Motani, 2003). Variation in tooth size and shape in Jurassic and Cretaceous ichthyosaurs is present but substantially reduced (Dick & Maxwell, 2015b; Fischer et al., 2016; Moon & Kirton, 2016).

Direct evidence of ichthyosaur diets comes from gut masses, supporting a mixed diet of fish and squid for many species and confirming dietary inferences made from adaptations of the body plan and dentition (Foffa et al., 2018). Cephalopod hooklets are commonly preserved in these fossil gut masses, in addition to varying amounts of fish bones and occasional terrestrial vertebrate remains suggesting opportunistic scavenging behaviour (Böttcher, 1989; Bürgin, 2000; Dick et al., 2016; Druckenmiller et al., 2014; Kear et al., 2003; Massare & Young, 2005; Pollard, 1968; Stinnesbeck et al., 2014). Bürgin (2000) reported remains of a nearly complete actinopterygian fish, *Euthynotus*, in the rib cage of a 2‐m‐long *Stenopterygius* from the Holzmaden Posidonienschiefer. In more detail, Dick et al. (2016) reported stomach contents from a series of *Stenopterygius quadriscissus* specimens from Holzmaden, comprising cephalopod remains (belemnitid, belemnoteuthid and phragmoteuthid hooklets), fish remains (at least four taxa: *Saurorhynchus*, *Dapedium*, pachycormid indet. and *Euthynotus*), and even an aborted *Stenopterygius* embryo. The relative proportions or volumes of different skeletal materials remaining in gut contents may be poor indicators of the relative importance of different organisms in the diet because some elements such as cephalopod hooklets likely survive in the acidic stomach fluids far longer than fish bones for example. However, these fossil finds show a good mix of cephalopod and fish remains, the former perhaps at an earlier stage in the digestive system.

### 4.3 Juvenile ichthyosaurs and dietary partitioning

By analogy with some modern shark species, it has been suggested that juvenile ichthyosaurs occupied sheltered shallow marine environments prior to a shift to a more pelagic lifestyle in adulthood, and the Strawberry Bank locality may reflect such an environment (Caine & Benton, 2011; Williams et al., 2015). BRSLI M1409 and BRLSI M1399, among the other ichthyosaurs of Strawberry Bank, have been identified as juveniles because of their small size relative to adult examples of the two species from coeval localities, most notably Holzmaden in Germany (Caine & Benton, 2011; Dick et al., 2016; Maxwell & Cortés, 2020; Miedema & Maxwell, 2019; Williams et al., 2015).

Such a dietary shift has been suggested in *S. quadriscissus* based on direct evidence of stomach contents (Dick et al., 2016). These authors reported nine identifiable categories of cephalopods, fishes and ichthyosaur remains in seven specimens, documenting a dietary shift from small, burst‐swimming fishes living in surface waters as the main food of juveniles to a diet exclusively of cephalopods that lived deeper in the water column in the adults. This dietary shift is confirmed by the dentitions of juvenile and adult *Stenopterygius*: juveniles have relatively long, sharp teeth indicative of Massare's (1987) Pierce II guild, whereas adults have reduced dentitions, and are sometimes toothless (Dick et al., 2016; Dick & Maxwell, 2015a; Massare, 1987). It is unknown if this is the case for *Hauffiopteryx*; in their revision of the German *Hauffiopteryx* material, Maxwell and Cortés (2020) identify juveniles and adults and confirm that the Strawberry Bank individuals are juveniles, but stomach contents have not been reported.

It is unknown whether this dietary shift from surface fishes to deeper‐water cephalopods occurred more widely among ichthyosaurs (Dick et al., 2016; Dick & Maxwell, 2015a). The allometric scaling of the cranium in ichthyosaurs throughout ontogeny is minimal in Jurassic ichthyosaurs, with changes throughout ontogeny limited to the shape and proportion of certain braincase elements and to the dentition (McGowan, 1973b; Miedema & Maxwell, 2019). The isometric growth of the ichthyosaur skull suggests that dietary changes related simply to size and increasing jaw forces rather than shifts in bite force or other changes in feeding mode. However, the increased jaw depth and posterior mechanical advantage in adult specimens of *Stenopterygius* compared to preserved foetuses suggest a transitory period soon after birth in which the jaws become relatively shorter and more robust (BCM, personal observation). Dietary niche partitioning between ichthyosaurs at all life stages likely allowed for the exploitation of a multitude of resources, as with extant cetaceans occupying similar ecological roles today. Following our analyses here of 2 three‐dimensional skulls of juvenile ichthyosaurs, it will be good in future to carry out similar studies on adults of the same, or similar, species. This, however, may be problematic as many localities yielding such specimens, including Holzmaden and the Yorkshire coast, certainly yield exceptionally preserved specimens sometimes with soft tissues, but the specimens tend to be flattened and so unsuitable for 3D functional analysis.

## 5 CONCLUSIONS

Niche partitioning between species and perhaps between life stages provides an explanation for the diversity of superficially similar ichthyosaurs throughout the Jurassic. Predators coexisting alongside one another and sharing a similar ecospace were able to remain diverse and had the potential to continue to diversify if fine‐scale dietary partitioning was possible through the division of food resources. We can see from the Strawberry Bank locality that a diverse array of prey resources was available, and that these two species of juvenile ichthyosaur successfully coexisted. Here, we performed FEA on ichthyosaurs for the first time and provide quantitative evidence that the robusticity of the rostrum in particular and the cranium more generally is important in feeding ability and in niche partitioning, with a more slender rostrum having less resistance to feeding stresses. We find that the more robust rostrum of *S. triscissus*, correlating to its more robust dentition and overall cranial morphology, and quantified by the stress distributions from the FEA, supports a feeding strategy of scavenging and hunting large fish or squid, while *H. typicus* was likely fishing for smaller and softer prey and relying more strongly on bite speed than on bite force while hunting. Future comparative FEA studies between similar ichthyosaur specimens, including the other juvenile specimens from Strawberry Bank, as well as related adults from other Early Jurassic localities, will shed further light on the potential for niche partitioning as a factor in ichthyosaur diversity.

## AUTHOR CONTRIBUTIONS

Benjamin C. Moon, Andre J. Rowe and Michael J. Benton designed and supervised the study, Sarah Jamison‐Todd carried out the analyses and wrote the first draft of the paper, Benjamin C. Moon provided code and training in 3D segmentation model‐building, Andre J. Rowe provided training and technical help with the FEA work. Matt Williams curated and loaned specimens and made them available for scanning and study. All authors contributed to drafting the manuscript. We also Kelly Vargas and Robert Wood for early segmentation work on BRLSI M1409. This project was supported by Leverhulme Trust Research Project Grant RPG‐2015‐126, NERC BETR grant NE/P013724/1 and ERC grant 788,203 (INNOVATION).

## Supporting information


Appendix S1

Appendix S2

## Data Availability

Data for this study are available in the University of Bristol Research Data Storage Facility (RDSF). (Please note that the data for this paper are not yet published and this information should not be shared without the express permission of the Author.)
