# Supplementary figures and images for: Dietary niche partitioning in Early Jurassic ichthyosaurs from Strawberry Bank
Source: J Anat. 2022 Sep 29;241(6):1409–23. doi: 10.1111/joa.13744 (PMC9644957; doi:10.1111/joa.13744)

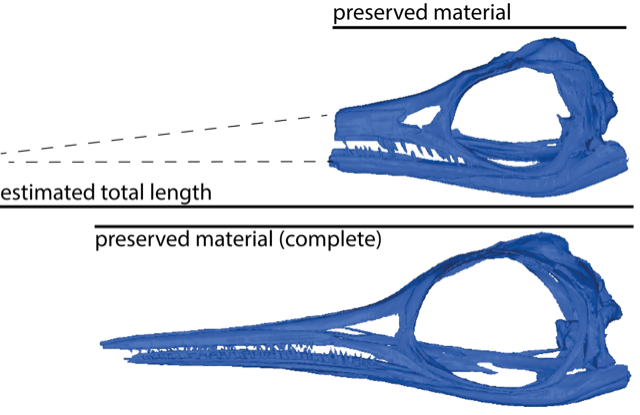

Supplement: Supplementary file 1 — Appendix S1 [file JOA-241-1409-s002.tiff]

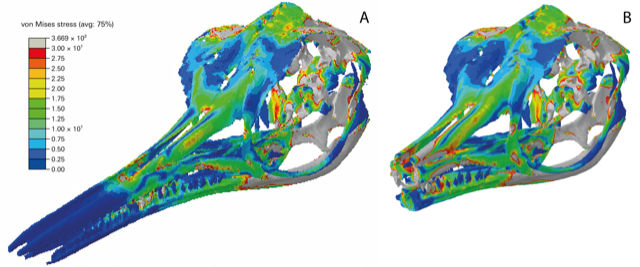

Supplement: Supplementary file 2 — Appendix S2 [file JOA-241-1409-s001.tiff]
